# Avelumab for the treatment of locally advanced or metastatic Merkel cell carcinoma—A multicenter real‐world experience in Israel

**DOI:** 10.1002/cam4.5890

**Published:** 2023-04-03

**Authors:** Itamar Averbuch, Ronen Stoff, Mor Miodovnik, Shlomit Fennig, Gil Bar‐Sela, Alexander Yakobson, Jonathan Daliot, Nethanel Asher, Eyal Fenig

**Affiliations:** ^1^ Davidoff Cancer Center, Rabin Medical Center, Sackler Faculty of Medicine Tel Aviv University Tel Aviv Israel; ^2^ Ella Institute for Immuno‐Oncology, Sheba Medical Center, Sackler, Faculty of Medicine Tel Aviv University Tel Aviv Israel; ^3^ Department of Dermatology, Sourasky Medical Center, Sackler Faculty of Medicine Tel Aviv University Tel Aviv Israel; ^4^ Institute of Oncology, Kaplan Medical Center, Faculty of Medicine Hebrew University Jerusalem Israel; ^5^ Cancer Center, Emek Medical Center, Rappaport Faculty of Medicine Israel Institute of Technology‐Technion Haifa Israel; ^6^ The Legacy Heritage Oncology Center & Dr. Larry Norton Institute, Soroka Medical Center, School of Medicine Ben Gurion University of the Negev Beer Sheva Israel

**Keywords:** avelumab, immunotherapy, Merkel cell carcinoma, real‐world experience

## Abstract

**Background:**

Merkel cell carcinoma (MCC) is a rare and aggressive malignancy of the skin, affecting predominantly the fair‐skinned older population exposed to high levels of ultraviolet light. Immune suppression is considered a significant risk factor. With the recent advances in the field of immunotherapy, the treatment paradigm for advanced MCC, traditionally based on chemotherapy, has largely shifted to anti‐PD‐L1 and PD‐1 agents such as avelumab and pembrolizumab, respectively. However, real‐world data remain sparse. The aim of this study was to assess real‐world evidence of the effectiveness of avelumab in a diverse group of patients with MCC in Israel.

**Methods:**

The electronic databases of five university hospitals in Israel were searched for all consecutive patients with MCC treated with at least one dose of avelumab in 2018–2022. Data on baseline, disease‐related, treatment‐related, and outcome parameters were collected and analyzed.

**Results:**

The cohort included 62 patients of whom 22% were immune‐suppressed. The overall response rate to avelumab was 59%. The median progression‐free survival was 8.1 months, and the median overall survival, 23.5 months, with no differences between immune‐competent and immune‐suppressed patients. Treatment was well tolerated; any‐grade toxicity developed in 34% of patients, and grade 3–4 toxicity, in 14%.

**Conclusions:**

Avelumab was found to be effective and safe for the treatment of advanced MCC in a diverse group of patients, including some with immune suppression. Further studies are warranted to evaluate the optimal sequence and duration of treatment and to assess the potential role of avelumab for earlier stages of MCC.

## Graphical abstract

Assessment of the effectiveness of avelumab in a diverse group of patients with MCC in multi‐center real‐world evidence from Israel. Avelumab was found to be effective and safe in a diverse group of patients, including some with immune suppression.

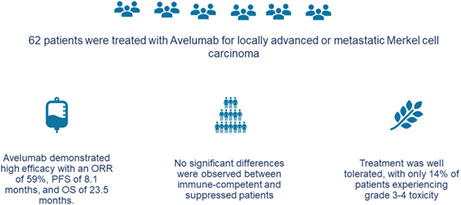

## 1 | INTRODUCTION

Merkel cell carcinoma (MCC) is an aggressive neuroendocrine malignancy of the skin.^1^ Its two leading known causes are exposure to UV lights or Merkel cell polyomavirus (MCPyV). It forms predominantly on sun‐exposed areas, mostly in older individuals.^2^ Although MCC is considered rare, recent publications report a consistent increase in incidence to 0.7 cases per 100,000 persons overall^3^, ^4^ and up to 1.6 cases per 100,000 persons in regions inhabited by fair‐skinned populations with high levels of ultraviolet light exposure.^5^, ^6^ In the United States, the number of incident cases is predicted to reach 3284 by 2025.^3^

The aggressive nature of MCC is manifested by its high risk of recurrence.^1^ Approximately 40% of patients present with advanced disease (involvement of the lymph nodes or distant metastases) already at diagnosis. The rate of MCC‐specific mortality ranges from 26% to 32%.^6^, ^7^, ^8^

MCC is considered a radiosensitive malignancy, and the main treatment modalities for local disease are surgery and radiation. Despite an in‐field control rate of 75%–85% with radiotherapy, metastatic disease eventually develops in most patients.^9^, ^10^ Systemic chemotherapy, for many years the treatment of choice for metastatic disease, was found to be associated with a high response rate but short duration of response.^11^ With the recent advances in immunotherapy, immune checkpoint inhibitors (ICIs) have become the preferred systemic treatment for patients without known contraindications.^12^ Avelumab, a programmed cell death ligand 1 (PD‐L1) blocking agent was the first FDA‐approved ICI for MCC. Approval was based on the phase II JAVELIN Merkel 200 trial wherein previously treated patients given avelumab showed an overall response rate (ORR) of 33%,^13^ 2‐year progression‐free survival (PFS) rate of 26%, and 2‐year overall survival (OS) rate of 36%.^14^ In the setting of first‐line metastatic MCC, the JAVELIN Merkel 200 part B yielded an ORR of 62.1%.^15^

To date, there are only a limited number of published real‐world studies on the use of avelumab in MCC.^16^ Real‐world data are of great importance because of the high incidence of MCC in immune‐suppressed patients, a population that is excluded from participating in clinical trials and has a worse prognosis than immune‐competent patients.^7^ Real‐world data from Israel are important because of the large number of fair‐skinned people in an area with significant ultraviolet exposure.

## 2 | METHODS

### 2.1 | Ethics

The study conformed to the standards required by the Declaration of Helsinki and was approved by the Institutional Review Board of each of the participating medical centers (0101‐07) which waived the need for informed consent.

### 2.2 | Patients and setting

A retrospective design was used. The study population consisted of all consecutive patients treated with avelumab for locally advanced or metastatic MCC at five leading academic medical centers in Israel between February 2018 and April 2022. Inclusion criteria included evaluable patients treated with at least one cycle of avelumab. Exclusion criteria were incomplete demographic or treatment related data, or participants which received avelumab as part of a pharma‐sponsored study.

### 2.3 | Clinical data

Patients were identified by database search, and data were extracted from their electronic medical records, as follows: demographics, imaging findings, tumor histology, disease stage, and Eastern Cooperative Oncology Group (ECOG) performance status (PS) at presentation. Response to treatment was categorized as complete response (CR), partial response (PR), stable disease (SD), progressive disease (PD), and mixed response, according to the assessment of the treating oncologist using the immune‐related Response Evaluation Criteria in Solid Tumors (iRECIST) criteria. Toxicity assessment was based on the Common Terminology Criteria for Adverse Events (CTCAE), version 5.0.^17^

### 2.4 | Outcome measures

The primary endpoint of the study was progression‐free survival (PFS). Secondary endpoints include overall survival (OS) and overall response rate (ORR).

### 2.5 | Treatment and follow‐up

Avelumab was administered at 10 mg/kg every 2 weeks. Dose changes were possible according to the decision of the attending oncologist. Routine follow‐ups were performed at the treating oncologist's discretion and included clinical follow‐up, physical examination and routine imaging. Blood tests for complete blood cells and chemistry including renal function, hepatic function, and thyroid function were performed before each cycle of treatment.

### 2.6 | Statistical analysis

All statistical analyses were generated using IBM© SPSS© software, version 25. Chi‐squared test of independence was performed to examine the relationship between subgroups of patients and their response rates to avelumab. Independent‐samples *t*‐test was used to examine the relationship between immunological status and time to maximum response. Log‐rank test was run to determine differences in PFS and OS between subgroups. A Cox regression model was used to quantify the hazard ratio for disease progression under avelumab based on immunological status and location of the primary tumor. *P* values <0.05 were considered significant.

## 3 | RESULTS

The cohort consisted of 62 evaluable patients with histologically proven MCC who received at least one cycle of avelumab. Data regarding the status of the MCPyV in the tumor biopsies was missing, as this is not routinely tested in Israel. Their demographic and baseline characteristics are outlined in Table 1. The median age at diagnosis was 74.5 years (range 37–95), and the male‐to‐ female ratio was 1.2:1. Primary tumor locations were the extremities in 23 patients (37.1%), head and neck in 15 (24.2%), trunk in 6 (10%), and unknown in 18 (29%). Fourteen patients (22.6%) were immune‐suppressed, including two solid‐organ (kidney) transplant recipients, nine patients with myeloproliferative diseases, and three with iatrogenic immune suppression. The ECOG PS score was 0 in 26 patients (42%), 1 in 12 patients (19%), and ≥2 in 12 patients (19%); data were unavailable for 12 patients (19%). At treatment initiation, 52 patients (84%) had stage IV metastatic disease and eight (13%) had locally advanced disease; data were unavailable for two patients (3%).

**TABLE 1:** Baseline characteristics of 62 patients with Merkel cell carcinoma treated with avelumab.

Characteristic	*N*
Age, median (range)	74.5 (37–95)
Sex	
Male	34 (55%)
Female	28 (45%)
Primary location	
Extremities	23 (37%)
Head and Neck	15 (24%)
Trunk	6 (10%)
Unknown	18 (29%)
Disease stage	
Metastatic	52 (84%)
Locally advanced	8 (13%)
Unknown	2 (3%)
ECOG PS	
0	26 (42%)
1	12 (19%)
2	8 (13%)
3	3 (5%)
4	1 (2%)
Unknown	12 (19%)

*Note*: All values are presented as *n* (%), unless otherwise stated.

Abbreviation: ECOG PS, Eastern Cooperative Oncological Group performance status.

Previous treatment data were available for 54 patients in the cohort, of whom 39 (72%) received avelumab as the first line of systemic treatment. Before initiation of avelumab, 31 patients (50%) received local treatment with radiation, surgery, or both. The median number of treatment cycles was 13.5 (range 1–118). The median follow‐up time was 12.75 months (range 0.2–55).

Response assessment was possible in 54 patients. The response patterns are shown in Table 2. The overall response rate (ORR) was 59.3%: 20 patients (37%) achieved CR and 12 (22%) achieved PR. Of the remainder, 6 patients (11%) had SD, 12 (22%) had PD, and 4 (7%) had a mixed response. The median PFS was 8.1 months (range 0–57) and the median OS was 23.5 months (range 3.2–101.2). The median time to maximal response was 4.25 months for patients who achieved CR and 3 months for patients who achieved PR. Among the responding patients, 12 (38%) needed local treatment (radiation or surgery) to preserve the response, and they continued to respond to avelumab thereafter.

**TABLE 2:** Response patterns in 54/62 patients with Merkel cell carcinoma treated with avelumab.

Response	*N* (%)
Overall response rate	32 (59%)
Partial response	12 (22%)
Complete response	20 (37%)
Stable disease	6 (11%)
Progressive disease	12 (22%)
Mixed response	4 (7%)

Comparison of immune‐suppressed and immune‐competent patients revealed no statistically significant differences in ORR (61% vs. 53.8%, *p* = 0.65), median PFS (15.2 months vs. 5.2 months, *p* = 0.4), or mean time to best response (4.72 vs. 4.64 months, *p* = 0.96). Among the immunosuppressed transplant patients, one patient (50%) achieved CR, while the other patient (50%) had maximum response to PD. Among the patients with hematological malignancy, three (33%) achieved CR, two (22%) achieved PR, two (22%) had SD, one (11%) had PD, and for one patient (11%), maximum response was not available. In three patients with the iatrogenic immune suppression group, one (33%) achieved CR, while two (67%) exhibited PD as their maximum response. The ORR in patients with an unknown primary was 65% compared to 56% in patients with a known primary (*p* = 0.581). There were no significant differences between these groups in median PFS (15.2 months vs. 8.1 months, *p* = 0.997) and median OS (41.6 vs. 39.6 months, *p* = 0.683).

Any‐grade toxicity was observed in 21 patients (34%) with 9 patients (14%) experiencing grade 3–4 toxicity. Six patients (10%) needed systemic treatment with glucocorticosteroids for a median duration of 3 months. Treatment was initiated as recommended by the American Society of Clinical Oncology (ASCO) guidelines for immune‐related adverse events.^18^ Four patients (6%) discontinued treatment due to toxicity. Complete data on the different toxicities are shown in Table 3. The occurrence of toxicity did not differ significantly between immunocompetent and immune‐suppressed patients (35% vs. 45%, *p* = 0.51). None of the organ transplant patients developed a rejection of the graft during treatment. In an immunosuppression etiology‐based analysis, one organ transplant patient (50%), and one patient (33%) from the iatrogenic immunosuppression group developed toxicity. Data regarding toxicity was available for six patients in the hematological malignancy group, three (50%) developed toxicity.

**TABLE 3:** Toxicities associated with avelumab treatment in 62 patients with Merkel cell carcinoma.

Toxicity	*N* (%)
Any grade	21 (34%)
Grade 3–4	9 (14%)
Thyroiditis	6 (10%)
Dermatitis	4 (6%)
Colitis	3 (5%)
Fatigue	3 (5%)
Hematological	2 (3%)
Hepatitis	2 (3%)
Miscellaneous ^a^	4 (6%)

^a^ Including neurological toxicity, arthralgia, chills, pneumonitis.

At the time of the data cutoff, 26 patients had died. The cause of death was MCC in 15 (58%) and non‐MCC‐related in four (15%); data were unavailable for seven patients (27%).

## 4 | DISCUSSION

MCC is a potentially fatal malignancy. Owing to its rarity, there is a shortage of randomized double‐blinded clinical trials examining new treatment regimens. The two main trials to date testing novel immunotherapy agents (anti‐PDL‐1 avelumab and anti PD‐1 pembrolizumab)^13^, ^19^ included only a small number of participants and excluded patients with preexisting immune suppression, a known risk factor for MCC.

As such, real‐world evidence in a more diverse patient population is lacking. We sought to collect data on all patients with MCC treated in five tertiary medical centers in Israel since 2018, when the Israel Ministry of Health included avelumab for reimbursement in the national health basket. A total of 62 patients were identified, of whom 22% were immune‐suppressed. The results revealed a real‐world response rate of 59%. This value is in line with the known data on response to first‐line treatment with avelumab, even though only 72% of the patients received avelumab as their first systemic line of therapy.

There was no difference in response by primary tumor location or between immune‐suppressed and immune‐competent patients. Due to the reasons above, data regarding ICI efficiency in immunosuppressed patients is scarce, so this study provides an essential addition to the existing efficacy data for avelumab. Due to the small sample size, a statistical analysis of efficacy in the subgroup of immune‐compromised patients was not feasible. Nevertheless, as seen in the descriptive presentation of the response, it does not seem that one specific group had a poorer outcome than the other. This is consistent with the previously published retrospective trial that assessed the efficiency of ICIs in MCC patients with a history of hematological malignancies, which showed that the presence of the hematological malignancy did not affect the treatment outcome.^20^

These findings support the choice to use avelumab as first‐line systemic therapy in all patients with MCC who did not have a contraindication for ICIs. Our overall disease‐control rate of 70% (CR + PR + SD) means that most of the patients derived some benefit from the treatment. This is particularly noteworthy considering the aggressive nature of MCC. Furthermore, the tendency of MCC to form dermal satellite tumors and its exquisite radiosensitivity allow for effective and safe local salvage treatments (radiotherapy or surgery) when the ICIs response is insufficient, as occurred in 38% of our patients. The addition of RT can explain the high response rate mentioned above. Several studies have demonstrated significant variability in the expression of immune checkpoint molecules and immune‐related genes within different areas of the same tumor.^21^, ^22^, ^23^ This heterogeneity can lead to suboptimal response rates that imaging may miss. However, RT has the potential to address these resistant areas in the tumor, converting cases that would have been classified as PD or SD into PR or CR. While data on this phenomenon in the context of MCC is limited, a previous study we published on cutaneous squamous cell carcinoma with PD‐1 inhibitors showed a similarly positive effect of RT.^24^ The potential combination of ICIs with radiotherapy is also intriguing in light of growing evidence on the ability of RT to indue improved immune response in other skin cancers such as melanoma.^25^, ^26^, ^27^

An impressive 37% of patients achieved CR, a rate higher than previously reported. Whether the CR is durable over the long term remains to be established, although data on response durability in MCC patients treated with anti‐PD1 (pembrolizumab) showed that 72.2% of the patients who responded, remained progression free at three years after treatment initiation.^19^ Whether this effect will be sustained for a longer duration of response as seen in other skin cancers such as melanoma is eagerly anticipated. Another important finding was the low toxicity of avelumab: 14% of patients had grade 3–4 toxicities, with only a 6% rate of treatment discontinuation. These results suggest a higher safety profile than previously reported in toxicity studies of PD‐1/PDL‐1 blockade for MCC (20%–30%),^15^, ^19^ especially given the nature of the population: elderly (mean age 74) and immune‐suppressed (22%). The strength of this study comes from its diverse population of both immune‐competent and immune‐suppressed patients in an area with high UV exposure, in contrast to most previously published series from geographical regions with significantly less UV exposure.

This study was limited by the retrospective design, absence of data on viral status in the pathological specimens, and the relatively small sample size. Further studies are required to elucidate predictive factors and optimal treatment duration, mainly for patients who achieve a durable response. Attention should be addressed in prospective studies to the role of ICIs at earlier disease stages, as either neoadjuvant or adjuvant treatment—an increasingly popular approach being used in other aggressive skin cancers.^28^, ^29^, ^30^, ^31^, ^32^ There is also a need for better treatment options for patients who are ineligible for ICI treatment and for those in whom the disease progresses on ICIs. In the era of precision medicine, the classification of MCC by primary location and patient immune status is still essential, yet inadequate in many cases. Multi‐omic based disease profiling (with novel biomarkers such as circulating microRNA) will likely involve both genetic and molecular analyses in the near future, leading to better treatment tailoring based on expression patterns within the tumor and the systemic circulation.^33^, ^34^ This approach might also help in assessing response to treatment, including the option of stopping treatment to those who have durable clinical and molecular responses. Future studies could assess the efficacy of avelumab based on the genetic and molecular characteristics of the disease.

In conclusion, this real‐world study shows that avelumab is effective in all subsets of patients with MCC, with a very well tolerated toxicity profile. Further studies are required to enhance treatment efficacy for patients with any‐stage MCC.

## AUTHOR CONTRIBUTIONS

**Itamar Averbuch:** Conceptualization (equal); data curation (equal); formal analysis (equal); writing – original draft (equal). **Ronen Stoff:** Conceptualization (equal); data curation (equal); formal analysis (equal); writing – original draft (equal). **Mor Miodovnik:** Data curation (equal). **Shlomit Fennig:** Data curation (equal). **Gil Bar‐Sela:** Data curation (equal). **Alexander Yakobson:** Data curation (equal). **Jonathan Daliot:** Data curation (equal). **Nethanel Asher:** Supervision (equal). **Eyal Fenig:** Supervision (equal); writing – original draft (equal); writing – review and editing (equal).

## FUNDING INFORMATION

No funding was procured for this work.

## CONFLICT OF INTEREST STATEMENT

The authors have no conflicts of interest.

## Data Availability

The data of this study are available from the authors upon reasonable request.
